# Blood biomarkers of various dietary patterns correlated with metabolic indicators in Taiwanese type 2 diabetes

**DOI:** 10.29219/fnr.v63.3592

**Published:** 2019-11-20

**Authors:** Meng-Chuan Huang, Chiao-I Chang, Wen-Tsan Chang, Yen-Ling Liao, Hsin-Fang Chung, Chih-Cheng Hsu, Shyi-Jang Shin, Kun-Der Lin

**Affiliations:** 1 Department of Nutrition and Dietetics, Kaohsiung Medical University Hospital, Kaohsiung, Taiwan; 2 Graduate Institute of Medicine and Department of Public Health and Environmental Medicine, School of Medicine, Kaohsiung Medical University, Kaohsiung, Taiwan; 3 Faculty of Medicine, College of Medicine, Kaohsiung Medical University, Kaohsiung, Taiwan; 4 Department of Surgery, Kaohsiung Medical University Hospital, Kaohsiung Medical University, Kaohsiung, Taiwan; 5 School of Public Health, University of Queensland, Brisbane, Queensland, Australia; 6 Institute of Population Health Sciences, National Health Research Institutes, Zhunan , Taiwan; 7 Division of Endocrinology and Metabolism, Department of Internal Medicine, Kaohsiung Medical University Hospital, Kaohsiung Medical University, Kaohsiung, Taiwan; 8 Department of Internal Medicine, Kaohsiung Municipal Ta-Tung Hospital, Kaohsiung Medical University, Kaohsiung, Taiwan

**Keywords:** dietary pattern, ferritin, n-3 PUFA, Taiwan, type 2 diabetes

## Abstract

**Background:**

Metabolic alterations correlate with adverse outcomes in type 2 diabetes. Dietary modification serves as an integral part in its treatment.

**Objective:**

We examined the relationships among dietary patterns, dietary biomarkers, and metabolic indicators in type 2 diabetes (*n* = 871).

**Design:**

Diabetic patients (*n* = 871) who provided complete clinical and dietary data in both 2008 and 2009 were selected from a cohort participating in a diabetic control study in Taiwan. Dietary data were obtained using a short, semiquantitative food frequency questionnaires, and dietary pattern identified by factor analysis. Multiple linear regressions were used to analyze the association between dietary biomarkers (ferritin, folate, and erythrocyte n-3 polyunsaturated fatty acids [n-3 PUFAs]) and metabolic control upon adjusting for confounders.

**Results:**

Three dietary patterns (high-fat meat, traditional Chinese food–snack, and fish–vegetable) were identified. Ferritin correlated positively with high-fat meat factor scores (*P* for trend <0.001). Erythrocyte n-3 PUFAs (eicosapentaenoic acid [EPA] + docosahexaenoic acid [DHA], n-3/n-6 PUFA ratio) correlated positively with fish–vegetable factor scores (all *P* for trends <0.001). Multiple linear regressions revealed a positive relationship between ferritin concentrations and fasting plasma glucose (FPG), hemoglobin A1c (HbA1c), and triglycerides, but a negative relationship with high-density lipoprotein cholesterol (HDL-C). Erythrocyte n-3 PUFA, EPA+DHA, and n-3/n-6 PUFA ratio were negatively linked to FPG, HbA1c, and triglycerides (all *P* < 0.05) and positively with HDL-C (though n-3/n-6 ratio marginally correlated).

**Conclusions:**

Ferritin and n-3 PUFA can serve as valid biomarkers for high-fat meat and fish–vegetable dietary patterns. Unlike ferritin, erythrocyte n-3 PUFA status was related to better glycemic and blood lipid profiles. Our results suggest that habitual consumption of diet pattern rich in fish and vegetables may contribute in part to a healthier metabolic profile in type 2 diabetes.

## Popular scientific summary

- Dietary modification serves as an integral part of treatments for diabetes.
- We find that habitual consumption of diet pattern rich in fish and vegetables may contribute in part to better glycemic and lipid control in type 2 diabetes.

In the past two decades, the prevalence of diabetes mellitus (DM) in developing countries has been increasing (1). Based on a report by the International Diabetes Federation (IDF), approximately 366 million adults were diagnosed with diabetes in 2011 and 552 million adults are expected to be diabetic by 2030 (2). A Taiwan nationwide survey (2000–2009) found that the overall incidence of DM rose from 0.76 to 0.93% within 10 years, equivalent to a 25% increase (3). Type 2 diabetes is a well-established risk factor for cardiovascular diseases, and patients with diabetes frequently have metabolic abnormalities, including hypertriglyceridemia, elevated low-density lipoprotein cholesterol (LDL-C), or high-density lipoprotein cholesterol (HDL-C) (4).

Epidemiological studies have associated some dietary patterns with risk of type 2 diabetes (5–8). Generally, these studies suggest that the healthy patterns, including higher intakes of vegetables and fruits, whole grains, fish, and low-fat dairy, may decrease diabetes risk and that the unhealthy patterns, including frequent intakes of sugars, processed and red meats, and fried foods, may increase the risk. Dietary pattern analysis has emerged as an alternative method for assessing habitual dietary exposures, can be used to assess diet–disease relationships, and provides appropriate dietary recommendations more likely to succeed in real circumstances (9). Although most of the studies using this dietary pattern analysis to investigate the effect of dietary pattern on risk of diabetes have focused on Caucasians in the United States (6) and Canada (8), some have focused on Asians in Japanese and Singapore (5, 10). We previously reported that fish–vegetable dietary patterns correlated significantly with diabetic kidney diseases in Taiwan (11, 12). As an alternative to questionnaire data, biomarkers can serve as a surrogate measurement of past dietary intake and can possibly serve as an alternative to food intake questionnaires (13–16). Dietary biomarkers in blood can reflect both dietary consumption and biological processes such as absorption, incorporation, and metabolism. For example, ferritin has been correlated with higher red meat intake (13), carotenoids and folate with fruit and vegetables intakes (14, 15), and n-3 polyunsaturated fatty acids (n-3 PUFAs) with fish and seafood intakes (16).

Till date, very few studies in Asia have explored dietary patterns, multiple dietary biomarkers, and metabolic parameters in type 2 diabetes simultaneously. This study was aimed to investigate the relationships between various dietary patterns, the dietary biomarkers (ferritin, folate, and erythrocyte n-3 PUFAs), and measurement of glycemic control (fasting plasma glucose [FPG] and hemoglobin A1c [HbA1c]) and lipid control (triglyceride and HDL-C) in patients with type 2 diabetes in Taiwan.

## Materials and methods

### Study design and subjects

The participants for this cross-sectional study were enrolled from the Diabetes Management through Integrated Delivery System (DMIDS) Project (NCT00288678 ClinicalTrial.gov) previously conducted in Taiwan. The DMIDS project was undertaken to investigate the effect of implementing case management for patients with type 2 diabetes in primary care clinics. Patients were recruited for that project between August 2003 and December 2005 and followed from the beginning of 2008 to the end of December 2012. Data, including anthropometric measurements, diabetic self-management status, food frequency questionnaires (FFQ) to assess habitual intakes of various foods, and clinical parameter measurements, were collected yearly. Patients were excluded if they were pregnant or on dialysis; if they had ever been diagnosed for type 1 diabetes, myocardial infarction or cerebrovascular accident, comorbid blindness or systemic disease (e.g. cancer); or if they had ever undergone foot amputation on Taiwan’s national insurance claim forms at baseline (2003–2005). The protocol of DMIDS project has been described previously (17).

For this cross-sectional study, a total of 871 type 2 diabetes patients aged 30–70 years, who provided complete dietary data in both 2008 and 2009 and who had provided complete demographic information and metabolic parameters measured in 2009, were included in the analysis. The study protocol was approved by the IRBs of both National Health Research Institutes/Taiwan and Kaohsiung Medical University Hospital. All patients provided written informed consent.

### Measurement of clinical parameters

Hypertension was defined in a subject who had a measurement of blood pressure of ≥140/90 mmHg. Biochemical analysis was performed on blood samples collected at least 8 h overnight fasting. FPG, cholesterol, triglyceride, HDL-C, and creatinine were analyzed using an auto-analyzer (Beckman Coulter LX20, Fullerton, CA, USA). HbA1c assay was performed using high-performance liquid chromatography (The G7, Tosoh Kobe, Japan). All clinical parameters were sent to a laboratory (Union Clinical Laboratory, Taipei, Taiwan) certified by the College of American Pathology and the US Commission on Office Laboratory Accreditation.

### Dietary assessment

Dietary data were collected by trained research assistants. A short, semiquantitative FFQ was used to assess dietary intake by face-to-face interviews in both 2008 and 2009. This FFQ was recently validated (18) for use with Taiwanese diabetes adults. The information surveyed included intake items or groups, frequency of food group consumption, and serving sizes. Participants were asked to report how frequently they consumed certain foods over the 12 months prior to answering the survey questions. The nine frequency options ranged from ‘almost never’ to ‘4–6 times per day.’ Portion sizes per meal or per day were recorded for some foods, such as milk, fruit, vegetable, meats, fish, egg, and Chinese staples. Factor analysis was used to identify the major dietary patterns derived from 44 food items or groups (18). Three dietary patterns were identified: (1) the high-fat meat dietary pattern, (2) the traditional Chinese food–snack pattern, and (3) the fish–vegetable pattern, similar to our previous report (11, 18). We identified dietary patterns in 2008 and 2009 first. Because similar patterns were obtained in 2008 and 2009, the factors scores were averaged, and those food items with factor loadings ≥0.2 (Supplementary Table 1) were considered to contribute importantly to any of the dietary pattern.

### Dietary biomarker analysis

Ferritin and folate were analyzed by chemiluminescence (ADVIA CentaurXPT; Siemens, Tarrytown, NY, USA). Erythrocyte lipids were extracted using a procedure modified from the Bligh and Dyer method (19). A derivative of fatty acid methyl esters (FAMEs) was extracted using boron trifluoride-methanol. The methylated fatty acids were quantified in a gas chromatography (Agilent 6890 N; Agilent Technologies, Santa Clara, CA, USA) equipped with a capillary column (30 m × 0.250 mm inner diameter × 0.25 mm film). The carrier gas was N2. The C17:0 (Sigma, St Louis, MO, USA) was added as internal standard. Twenty individual fatty acids were identified by comparing findings with known standards. The concentration of each fatty acid was quantified as a percentage of total area under the peaks (weight %) (17). Ferritin and erythrocyte fatty acids were analyzed in both 2008 and 2009, and their mean concentrations were used for statistical analysis. Due to the limited availability of plasma specimen, folate was only measured for 2009.

### Statistical analysis

Descriptive data were expressed as mean ± SD for continuous or *N* (%) for categorical variables. Chi-square tests and one-way ANOVA were used to examine the differences in categorical and continuous variables among three dietary pattern score quartiles. Simple linear regression was used to analyze metabolic parameters and dietary biomarkers across dietary pattern quartiles. Multiple linear regression analysis was used to examine the independent association between dietary biomarkers and FPG, HbA1c, triglycerides, and HDL-C after adjusting for confounders. We adjusted for age (<65, ≥65 years), gender, body mass index (<24.0, ≥24.0 kg/m^2^), education (≤6, >6 years), diabetes duration (<7, ≥7 years), energy intake (continuous), hypertension (yes, no), and hypoglycemic agent use (yes, no). Hypolipidemic agents use (yes, no) was further adjusted as the outcome variables were TG and HDL-C. All statistical operations were performed using IBM SPSS Version 22.0 (Chicago, IL, USA). A two-tailed *P*-value <0.05 was considered significant.

## Results

### Patients’ characteristics analyzed by dietary pattern

As can been seen in Table 1, it presents the relations between study population characteristics across quartile of the three dietary pattern factor scores. The higher the quartile level of high-fat meat pattern, the greater the proportions of patients less than 65 years old, being males, patients with more than 6 years of education, and patients who had the habits of smoking and no drinking (all *P* < 0.05). The higher the traditional Chinese food–snack diet score quartile, the greater the proportions of patients aged less than 65 years old, patients with more than 6 years of education, and patients with diabetes more than 7 years (all *P* < 0.05). Moreover, the higher the fish–vegetable diet factor score quartile, the greater the proportions of patients more than 65 years old, less than 6 years of education, and patients who did not smoke (*P* < 0.05).

**Table 1:** Correlations between quartiles of dietary patterns and demographic characteristic and metabolic parameters in type 2 diabetes (*N* = 871)

Patient characteristics	High-fat meat diet			Traditional Chinese food–snack diet			Fish–vegetable diet		
	Q1	Q4	*P*^a^	Q1	Q4	*P*^a^	Q1	Q4	*P*^a^
Age (years)									
<65	116 (53.5)	175 (80.6)	<0.001	112 (51.6)	163 (74.8)	<0.001	152 (70.0)	121 (55.8)	0.012
≥65	101 (46.5)	42 (19.4)		105 (48.4)	55 (25.2)		65 (30.0)	96 (44.2)	
Gender									
Male	58 (26.7)	151 (69.6)	<0.001	103 (47.5)	98 (45.0)	0.935	113 (52.1)	100 (46.1)	0.155
Female	159 (73.3)	66 (30.4)		114 (52.5)	120 (55.0)		104 (47.9)	117 (53.9)	
Education year									
≤6	139 (64.1)	73 (33.6)	<0.001	155 (71.4)	100 (45.9)	<0.001	104 (47.9)	125 (57.6)	0.033
>6	78 (35.9)	144 (66.4)		62 (28.6)	118 (54.1)		113 (52.1)	92 (42.4)	
BMI (kg/m^2^)									
<24	74 (34.1)	64 (29.5)	0.773	67 (31.2)	66 (30.4)	0.936	65 (30.1)	69 (31.9)	0.941
≥24	143 (65.9)	153 (70.5)		148 (68.8)	151 (69.6)		151 (69.9)	147 (68.1)	
Diabetes duration (years)									
<7	64 (29.5)	83 (38.8)	0.103	48 (22.6)	79 (36.7)	0.006	73 (33.6)	61 (28.4)	0.379
≥7	153 (70.5)	131 (61.2)		168 (77.4)	135 (63.3)		144 (66.4)	154 (71.6)	
Smoking									
Yes	30 (13.8)	117 (53.9)	<0.001	64 (29.8)	76 (34.9)	0.410	85 (39.4)	76 (35.0)	0.003
No	187 (86.2)	100 (46.1)		151 (70.2)	142 (65.1)		131 (60.6)	141 (65.0)	
Alcohol drinking									
Yes	12 (5.5)	58 (26.7)	<0.001	20 (9.3)	30 (13.8)	0.225	25 (11.6)	33 (15.2)	0.726
No	205 (94.5)	159 (73.3)		195 (90.7)	188 (86.2)		191 (88.4)	184 (84.8)	

a Chi-square tests were used to examine differences in quartile 1 to quartile 4.

Data were expressed as *N* (%). *P <* 0.05 was considered significant.

BMI, Body mass index.

### Metabolic parameters and dietary biomarkers analyzed by dietary pattern

Metabolic parameters and dietary biomarkers were analyzed against dietary pattern score quartiles (Table 2). The high-fat meat dietary pattern scores were positively correlated with diastolic blood pressure (DBP), FPG, HbA1c, ferritin, n-3 PUFAs, eicosapentaenoic acid (EPA) + docosahexaenoic acid (DHA), and n-3/n-6 PUFA ratio, and negatively correlated with HDL-C and folate in a dose responsive manner (all *P* for trends <0.05). The traditional Chinese food–snack pattern was positively associated with triglycerides, and negatively associated with n-3 PUFA, EPA+DHA, and n-3/n-6 PUFA ratio (all *P* for trends <0.05). The fish–vegetable pattern was significantly correlated with reduced concentrations of serum triglyceride, and increased levels of n-3 PUFA, EPA+DHA, and n-3/n-6 PUFA ratio (all *P* for trends <0.05) (Table 2).

**Table 2:** Metabolic parameters in 871 patients with type 2 diabetes analyzed by quartile levels of dietary pattern score

	Quartiles of dietary patterns				*P*^a^	*P*-trend^b^
	Q1	Q2	Q3	Q4		
**High-fat meat diet**						
Metabolic parameters						
SBP (mmHg)	135.8 ± 19.1	136.3 ± 20.0	134.5 ± 17.7	135.9 ± 16.4	0.766	0.659
DBP (mmHg)	76.1 ± 10.4	76.7 ± 11.1	78.9 ± 10.4	81.9 ± 9.3	<0.001	<0.001
Fasting plasma glucose (mg/dL)	146.6 ± 50.5	145.2 ± 51.8	153.4 ± 56.9	161.2 ± 60.0	0.009	<0.001
Hemoglobin A1c (%)	7.8 ± 1.5	7.7 ± 1.6	7.9 ± 1.6	8.0 ± 1.6	0.181	0.007
Triglyceride (mg/dL)	168.7 ± 108.9	149.7 ± 76.4	149.1 ± 115.2	181.2 ± 146.2	0.007	0.250
Cholesterol (mg/dL)	194.7 ± 39.1	196.9 ± 37.4	192.9 ± 35.7	194.7 ± 39.3	0.745	0.720
HDL-C (mg/dL)	49.1 ± 12.5	49.8 ± 12.1	49.4 ± 12.3	44.9 ± 11.4	<0.001	0.003
Dietary biomarkers						
Ferritin (ng/mL)	165.8 ± 150.4	172.2 ± 131.7	190.6 ± 168.6	247.5 ± 222.5	<0.001	<0.001
Folate (ng/mL)	11.6 ± 12.3	8.5 ± 6.4	8.7 ± 5.8	8.7 ± 10.2	0.002	0.006
n-3 PUFA	9.5 ± 2.3	10.2 ± 1.4	10.3 ± 1.6	10.7 ± 1.8	<0.001	<0.001
EPA+DHA	7.2 ± 2.1	7.9 ± 1.3	8.0 ± 1.4	8.3 ± 1.6	<0.001	<0.001
n-3/n-6 PUFA ratio	0.31 ± 0.09	0.34 ± 0.06	0.34 ± 0.7	0.34 ± 0.7	<0.001	<0.001
**Traditional Chinese food–snack diet**						
Metabolic parameters						
SBP (mmHg)	137.5 ± 18.9	135.2 ± 18.2	134.9 ± 19.0	135.0 ± 17.2	0.378	0.026
DBP (mmHg)	77.6 ± 10.2	77.8 ± 11.4	78.2 ± 10.9	80.1 ± 9.4	0.046	0.057
Fasting plasma glucose (mg/dL)	154.9 ± 58.3	143.4 ± 45.5	147.4 ± 52.7	160.7 ± 61.8	0.005	0.370
Hemoglobin A1c (%)	8.0 ± 1.6	7.6 ± 1.4	7.8 ± 1.6	8.1 ± 1.6	0.014	0.539
Triglyceride (mg/dL)	149.3 ± 75.5	151.9 ± 86.5	169.0 ± 133.9	178.2 ± 145.9	0.024	0.022
Cholesterol (mg/dL)	194.9 ± 36.7	193.4 ± 37.5	194.4 ± 38.3	196.5 ± 39.2	0.864	0.936
HDL-C (mg/dL)	48.9 ± 11.6	49.1 ± 13.2	47.9 ± 12.7	47.4 ± 11.3	0.410	0.051
Dietary biomarkers						
Ferritin (ng/mL)	191.7 ± 162.8	185.4 ± 131.4	201.5 ± 198.0	198.9 ± 199.7	0.806	0.695
Folate (ng/mL)	8.8 ± 6.2	10.1 ± 12.9	9.3 ± 9.8	9.2 ± 6.2	0.577	0.848
n-3 PUFA	11.1 ± 1.7	10.3 ± 1.7	10.1 ± 1.7	9.5 ± 1.9	<0.001	<0.001
EPA+DHA	8.7 ± 1.4	8.0 ± 1.5	7.7 ± 1.5	7.2 ± 1.8	<0.001	<0.001
n-3/n-6 PUFA ratio	0.37 ± 0.07	0.34 ± 0.07	0.32 ± 0.07	0.29 ± 0.07	<0.001	<0.001
**Fish–vegetable diet**						
Metabolic parameters						
SBP (mmHg)	135.4 ± 18.5	136.0 ± 18.5	134.5 ± 17.9	136.7 ± 18.4	0.629	0.700
DBP (mmHg)	78.2 ± 10.8	78.2 ± 10.2	78.1 ± 10.7	79.2 ± 10.5	0.649	0.577
Fasting plasma glucose (mg/dL)	148.4 ± 52.7	154.2 ± 56.7	154.3 ± 54.1	149.4 ± 57.4	0.554	0.901
Hemoglobin A1c (%)	7.9 ± 1.5	7.9 ± 1.7	7.9 ± 1.4	7.8 ± 1.6	0.989	0.397
Triglyceride (mg/dL)	176.9 ± 132.5	168.2 ± 138.8	153.1 ± 87.7	150.4 ± 89.9	0.050	<0.001
Cholesterol (mg/dL)	196.1 ± 39.1	195.2 ± 36.9	193.0 ± 35.9	194.9 ± 39.5	0.855	0.399
HDL-C (mg/dL)	47.3 ± 11.7	48.8 ± 12.6	48.9 ± 12.0	48.3 ± 12.6	0.552	0.128
Dietary biomarkers						
Ferritin (ng/mL)	197.4 ± 180.8	199.9 ± 202.9	186.6 ± 162.5	193.3 ± 149.5	0.888	0.350
Folate (ng/mL)	9.7 ± 13.1	9.1 ± 6.4	8.9 ± 5.9	9.6 ± 9.5	0.799	0.892
n-3 PUFA	9.6 ± 1.7	9.8 ± 1.7	10.1 ± 1.6	11.3 ± 1.8	<0.001	<0.001
EPA+DHA	7.3 ± 1.5	7.5 ± 1.5	7.8 ± 1.4	8.9 ± 1.6	<0.001	<0.001
n-3/n-6 PUFA ratio	0.31 ± 0.07	0.32 ± 0.07	0.33 ± 0.06	0.37 ± 0.07	<0.001	<0.001

a ANOVA was used to examine differences among quartile 1, 2, 3, and 4 levels.

b Simple linear regression was used to examine the trend between metabolic parameters or dietary biomarkers against four quartile levels of three major dietary patterns.

Data were expressed as mean ± SD and *P <* 0.05 was considered significant.

### Independent association between dietary biomarkers and metabolic parameters

As shown in Table 3, a summary of the results of our multiple liner regression analyses, ferritin was correlated positively with FPG, HbA1c, and triglycerides, and correlated negatively with HDL-C (*P* < 0.001). Folate was correlated positively with FPG (*P* < 0.05), but was not correlated with HbA1c. Three erythrocyte n-3 fatty acid biomarkers (n-3 PUFA, EPA+DHA, and n-3/n-6 PUFA ratio), however, seemed to be linked negatively to FPG, HbA1c, and triglycerides and positively to HDL-C, though n-3/n-6 PUFA ratio was only marginally associated with HDL-C (*P* = 0.054) (Table 3).

**Table 3:** Association between dietary biomarkers and metabolic parameters after adjustment for confounders by multiple liner regression

	Ferritin (ng/mL)		Folate (ng/mL)		n-3 PUFA		EPA+DHA		n-3/n-6 PUFA ratio	
	*β* (SE)	*P*	*β* (SE)	*P*	*β* (SE)	*P*	*β* (SE)	*P*	*β* (SE)	*P*
Fasting plasma glucose (mg/dL)										
Crude model	0.055 (0.011)	<0.001	0.465 (0.211)	0.028	−1.737 (1.226)	0.157	−1.924 (1.347)	0.154	−49.002 (30.307)	0.106
Model 1^a^	0.061 (0.012)	<0.001	0.432 (0.210)	0.040	−2.903 (1.309)	0.027	−3.124 (1.431)	0.029	−64.715 (31.980)	0.043
Model 2^b^	0.062 (0.012)	<0.001	0.456 (0.212)	0.032	−2.943 (1.310)	0.025	−3.224 (1.432)	0.025	−66.494 (31.947)	0.038
Hemoglobin A1c × 10 (%)										
Crude model	0.007 (0.003)	0.037	0.067 (0.060)	0.263	−0.877 (0.343)	0.011	−0.895 (0.377)	0.018	−25.211 (8.464)	0.003
Model 1^a^	0.007 (0.003)	0.027	0.056 (0.060)	0.351	−1.308 (0.365)	<0.001	−1.333 (0.399)	0.001	−33.048 (8.901)	<0.001
Model 2^b^	0.008 (0.003)	0.020	0.068 (0.061)	0.265	−1.296 (0.367)	<0.001	−1.336 (0.401)	0.001	−32.991 (8.927)	<0.001
Triglyceride (mg/dL)										
Crude model	0.213 (0.021)	<0.001	0.699 (0.419)	0.095	−12.02 8 (2.541)	<0.001	−13.104 (2.794)	<0.001	−177.51 (63.629)	0.005
Model 1^a^	0.231 (0.022)	<0.001	0.800 (0.424)	0.060	−14.300 (2.772)	<0.001	−15.390 (3.032)	<0.001	−200.81 (68.704)	0.004
Model 3^c^	0.240 (0.022)	<0.001	0.637 (0.424)	0.134	−15.952(2.766)	<0.001	−17.064 (3.032)	<0.001	−236.70 (68.851)	0.001
HDL-C (mg/dL)										
Crude model	−0.015 (0.002)	<0.001	0.066 (0.047)	0.162	0.469 (0.276)	0.090	0.545 (0.303)	0.073	8.407 (6.833)	0.219
Model 1^a^	−0.010 (0.002)	<0.001	0.006 (0.046)	0.888	0.704 (0.284)	0.014	0.736 (0.311)	0.018	13.245 (6.961)	0.058
Model 3^c^	−0.010 (0.002)	<0.001	0.011 (0.046)	0.819	0.726 (0.290)	0.013	0.756 (0.318)	0.018	13.723 (7.113)	0.054

a Model 1 was adjusted for demographic characteristic (age, gender, body mass index, diabetes duration, education, and energy intake).

b Model 2 was adjusted for confounders in model 1 plus hypertension and hypoglycemic agents use.

c Model 3 was adjusted for confounders in model 1 plus hypertension, hypolipidemic, and hypoglycemic agents use.

Data were expressed as beta (SE) and *P <* 0.05 was considered significantly different.

## Discussion

This investigation, the first in Taiwan to study the possible association between dietary biomarkers of major dietary patterns and metabolic profiles among type 2 diabetes, found positive relationships between concentrations of the dietary biomarker ferritin and the metabolic parameters FPG, HbA1C, and triglycerides, and a negative relationship between this marker and HDL-C. It also linked three erythrocyte n-3 PUFA biomarkers (n-3 PUFA, EPA+DHA, n-3/n-6 PUFA ratio) to increases in HDL-C and decreases in FPG, HbA1c, and triglycerides.

One recent meta-analysis of observational studies has found an independent correlation between high serum ferritin and risks of metabolic syndrome (20). Another meta-analysis including cross-sectional and prospective studies revealed that subjects with highest quartile of ferritin were at significantly higher relative risk of having or developing type 2 diabetes compared to those in the lowest quartile (21). In addition, one study comparing ferritin concentrations in healthy controls with subjects with impaired glucose tolerance and normal glucose-tolerant first-degree relatives of type 2 diabetes patients, found increases in both study subject groups and suggested that overexpression of ferritin occurs before elevations in plasma glucose (22). Similar to those observed in healthy populations (23), in subjects with type 2 diabetes, increased ferritin concentrations have been associated with worsened triglyceride, blood pressure, and liver enzyme profiles (24). Considered together, the results of the above studies (20–24) and those of the current investigation all demonstrated that there is a correlation between increased ferritin concentrations and worsening of several metabolic parameters, including blood glucose, triglycerides, and HDL-C in type 2 diabetes. Based on human studies, iron overload has been postulated to affect insulin secretion and resistance, leading to decreased insulin sensitivity (25) and contributing to earlier complications in diabetes (26).

Red meat or processed meat intake has been correlated with overexpression of ferritin, insulin resistance, and hyperglycemia in healthy populations in the United States (13) and Sweden (27) as well as in European subjects with diabetes (28). The relationship between these adverse metabolic conditions and the Western dietary pattern, which is typically high in red meat or fatty meat, may be mediated through several mechanisms, including intake of saturated fat, foods with different glycemic loads, nitrites, and heme iron (13). In mouse models, iron excess has been shown to cause increased beta-cell oxidative stresses and decreased insulin secretory secondary to beta-cell apoptosis, loss of beta-cell mass, and desensitization of glucose-induced insulin secretion (29). Iron can also interfere with insulin extraction in the liver, and thereby contribute to peripheral hyperinsulinemia (30). Insulin has been postulated to induce a rearrangement of transferrin receptors to the cell surface where they mediate uptake of extracellular iron, activation of oxidative stress, and release of inflammatory cytokines in the subendothelial space, which, in turn, upregulate transcription and translation of ferritin mRNA in macrophages (31). Higher vascular iron deposition and accompanying superoxide release were observed in mice fed high-fat diet with iron administration (32). This observation offers a plausible explanation of the link between high-fat diets and development of cardiovascular disease, possibly occurring through fatty acid–iron extracellular oxidative stress and subsequent membrane oxidative injury. Based on the above observation, insulin resistance seems to be one pathological basis for the development of diabetes as well as risk of cardiovascular diseases. Excess body iron can impose oxidative injury, which has been reported to be correlated with several cardiovascular risk factors including dyslipidemia.

One prospective study (33) used 10 dietary biomarkers to explore the relationship between diet and type 2 diabetes independent of typical risk factors for type 2 diabetes and cardiovascular disease. Two biomarkers of fish intake, EPA (*P* = 0.041) and 3-carboxy-4-methyl-5-propyl-2-furanpropanoic acid (*P* = 0.002), were found to be inversely associated with future development of type 2 diabetes (33). Plasma levels of the long chain n-3 PUFA, EPA, have been correlated with decreases in fasting glucose and increases in HDL-C in patients with type 2 diabetes (34), and n-3 PUFA has been correlated with decreases in triglycerides (35). Increases in some n-3 erythrocyte fatty acids have been correlated with the consumption of certain foods including fish (36). One randomized crossover trial has reported that a 4-week fish-based diet intervention reduced blood triglyceride concentrations (16). We observed a significant association between higher fish–vegetable factor scores and higher erythrocyte n-3 PUFA levels (n-3 PUFA, EPA+DHA, n-3/n-6 PUFA ratio). Our study, as well as those of others (16, 37), found a clear association between frequent fish intake and/or subsequent increases in blood n-3 PUFA and better glucose and lipid profiles in patients with type 2 diabetes. However, a significant and positive association between fish consumption and fasting blood glucose were also observed (38). Omega-3 has been found to have a beneficial effect on insulin sensitivity and glucose metabolism in obese and insulin-resistant animals (39). However, in humans this relationship may be more complicated, and there is a need to also consider the effect of age, disease progression, and medication use as well as underlying comorbidities.

One systematic review of intervention studies found that consuming oily fish led to significant improvements in two important biomarkers of cardiovascular risk, such as triglycerides and HDL levels (40). Epidemiological studies have also found an association between high fish consumption and reduced risk of stroke (41) and heart disease (42, 43). However, another review of seven randomized clinical trials (RCTs) and one single-arm study (2002–2010) concluded that capsule supplementation with n-3 PUFA did not improve glycemic control (44). In contrast, that review cited one prospective cohort study (45) focused on whole food omega-3 intake and observed an inverse relationship between baseline marine omega-3 fatty acid intake and triglycerides. The American Diabetes Association (ADA) recommends that people with diabetes eat fish, particularly fatty fish, at least 2 times (two servings) per week to obtain optimal levels of long-chain n-3 PUFA (EPA and DHA) because observational studies have found these fatty acids to have beneficial effects on lipoproteins and positive health outcomes (46).

Results of a meta-analysis study including 763 participants with metabolic diseases from 16 randomized controlled trials revealed that folate supplementation significantly leads to decreases in insulin levels and HOMA-IR, but does not seem to affect FPG and HbA1c levels (47). In the current study, plasma folate concentrations were correlated positively and significantly with FPG, but not with other metabolic parameters. Even though there has been evidences showing increased blood folate and better glycemic control in diabetic patients (47), we did not have clear evidence to confirm previous findings. There are many factors could have affected the relationships, such as diabetes duration, medication use, and comorbidities. Thus, relations between blood folate and glycemic control may need further investigations.

The study was strengthened by the administration of face-to-face interviews by trained research personnel using a validated semi-quantitative FFQ (18) and two consecutive measurements of dietary biomarkers (except for folate) in blood were performed instead of single-time-point measurement. Thus, variabilities derived from assessment of food consumption can be minimized. Correlations between food consumption pattern and dietary biomarkers can, thus, be ascertained. This study has some limitations. One limitation was that our study sample size was small and may not be applied to other Asian populations with different eating habits. Another limitation of this study was that it had a cross-sectional design, and thus we could not confidently determine a cause–effect relationship between dietary patterns, dietary biomarkers, and metabolic profiles. Still another limitation was that there may be other unmeasured or residual confounding effects, not ruled out by the confounders we adjusted for in our multivariable regression analyses.

## Conclusions

In conclusion, our results suggest that ferritin and n-3 PUFA in blood can serve as valid dietary biomarkers for high-fat meat and fish–vegetable dietary patterns, respectively. Unlike ferritin, erythrocyte n-3 PUFA indicators were associated with better glycemic and blood lipid levels. Thus, habitual consumption of a fish–vegetable diet may help improve metabolic profiles in people with type 2 diabetes.

## Supplementary Material

Blood biomarkers of various dietary patterns correlated with metabolic indicators in Taiwanese type 2 diabetes

## References

[1] Yang G, Kong L, Zhao W, Wan X, Zhai Y, Chen LC, et al Emergence of chronic non-communicable diseases in China. Lancet 2008; 372(9650): 1697–705. doi: 10.1016/S0140-6736(08)61366-5. 18930526

[2] Whiting DR, Guariguata L, Weil C, Shaw J IDF diabetes atlas: global estimates of the prevalence of diabetes for 2011 and 2030. Diabetes Res Clin Pract 2011; 94(3): 311–21. doi: 10.1016/j.diabres.2011.10.029. 22079683

[3] Jiang YD, Chang CH, Tai TY, Chen JF, Chuang LM Incidence and prevalence rates of DM in Taiwan: analysis of the 2000–2009 Nationwide Health Insurance database. J Formos Med Assoc 2012; 111(11): 599–604. doi: 10.1016/j.jfma.2012.09.014. 23217595

[4] Quispe R, Martin SS, Jones SR Triglycerides to high-density lipoprotein- cholesterol ratio, glycemic control and cardiovascular risk in obese patients with type 2 diabetes. Curr Opin Endocrinol Diabetes Obes 2016; 23(2): 150–6. doi: 10.1097/MED.0000000000000241. 26863278

[5] Erber E, Hopping BN, Grandinetti A, Park SY, Kolonel LN, Maskarinec G Dietary patterns and risk for diabetes: the multiethnic cohort. Diabetes Care 2010; 33(3): 532–8. doi: 10.2337/dc09-1621. 20007939 PMC2827503

[6] Malik VS, Fung TT, van Dam RM, Rimm EB, Rosner B, Hu FB Dietary patterns during adolescence and risk of type 2 diabetes in middle-aged women. Diabetes Care 2012; 35(1): 12–18. doi: 10.2337/dc11-0386. 22074723 PMC3241320

[7] Nettleton JA, Steffen LM, Ni H, Liu K, Jacobs DR Jr Dietary patterns and risk of incident type 2 diabetes in the Multi-Ethnic Study of Atherosclerosis (MESA). Diabetes Care 2008; 31(9): 1777–82. doi: 10.2337/dc08-0760. 18544792 PMC2518344

[8] Reeds J, Mansuri S, Mamakeesick M, Harris SB, Zinman B, Gittelsohn J, et al Dietary Patterns and Type 2 Diabetes Mellitus in a First Nations Community. Can J Diabetes 2016; 40(4): 304–10. doi: 10.1016/j.jcjd.2016.05.001. 27374251

[9] Newby PK, Tucker KL Empirically derived eating patterns using factor or cluster analysis: a review. Nutr Rev 2004; 62(5): 177–203. doi: 10.1301/nr.2004.may.177-203. 15212319

[10] Odegaard AO, Koh WP, Butler LM, Duval S, Gross MD, Yu MC, et al Dietary patternsand incident type 2 diabetes in chinese men and women: the Singapore Chinese health study. Diabetes Care 2011; 34(4): 880–5. doi: 10.2337/dc10-2350. 21330641 PMC3064045

[11] Chung HF, Hsu CC, Mamun AA, Long KZ, Huang YF, Shin SJ, et al Dietary patterns, dietary biomarkers, and kidney disease in patients with type 2 diabetes: a repeated-measure study in Taiwan. Asia Pac J Clin Nutr. 2018; 27(2): 366–74. doi: 10.6133/apjcn.042017.15. 29384324

[12] Hsu CC, Jhang HR, Chang WT, Lin CH, Shin SJ, Hwang SJ, et al Associations between dietary patterns and kidney function indicators in type 2 diabetes. Clin Nutr 2014; 33(1): 98–105. doi: 10.1016/j.clnu.2013.04.010. 23706976

[13] Fung TT, Rimm EB, Spiegelman D, Rifai N, Tofler GH, Willett WC, et al Association between dietary patterns and plasma biomarkers of obesity and cardiovascular disease risk. Am J Clin Nutr 2001; 73(1): 61–7. doi: 10.1093/ajcn/73.1.61. 11124751

[14] Couillard C, Lemieux S, Vohl MC, Couture P, Lamarche B Carotenoids as biomarkers of fruit and vegetable intake in men and women. Br J Nutr 2016; 116(7): 1206–15. doi: 10.1017/S0007114516003056. 27572625

[15] Lee JE, Chan AT Fruit, vegetables, and folate: cultivating the evidence for cancer prevention. Gastroenterology 2011; 141(1): 16–20. doi: 10.1053/j.gastro.2011.05.020. 21620843 PMC3391696

[16] Kondo K, Morino K, Nishio Y, Kondo M, Nakao K, Nakagawa F, et al A fish- based diet intervention improves endothelial function in postmenopausal women with type 2 diabetes mellitus: a randomized crossover trial. Metabolism 2014; 63(7): 930–40. doi: 10.1016/j.metabol.2014.04.005. 24850465

[17] Chung HF, Long KZ, Hsu CC, Al Mamun A, Jhang HR, Shin SJ, et al Association of n-3 polyunsaturated fatty acids and inflammatory indicators with renal function decline in type 2 diabetes. Clin Nutr 2015; 34(2): 229–34. doi: 10.1016/j.clnu.2014.02.009 24721145

[18] Huang MC, Lin KD, Chen HJ, Wu YJ, Chang CI, Shin SJ, et al Validity of a short food frequency questionnaire assessing macronutrient and fiber intakes in patients of Han Chinese descent with type 2 diabetes. Int J Environ Res Public Health 2018; 15(6): 1142–55. doi: 10.3390/ijerph15061142. PMC6025601 29857595

[19] Bligh EG, Dyer WJ A rapid method of total lipid extraction and purification. Can J Biochem Physiol 1959; 37(8): 911–17. doi: 10.1139/o59-099. 13671378

[20] Abril-Ulloa V, Flores-Mateo G, Sola-Alberich R, Manuel-y-Keenoy B, Arija V Ferritin levels and risk of metabolic syndrome: meta-analysis of observational studies. BMC Public Health 2014; 14: 483. doi: 10.1186/1471-2458-14-483. 24884526 PMC4042131

[21] Orban E, Schwab S, Thorand B, Huth C Association of iron indices and type 2 diabetes: a meta-analysis of observational studies. Diabetes Metab Res Rev 2014; 30(5): 372–94. doi: 10.1002/dmrr.2506. 24327370

[22] Ren Y, Tian H, Li X, Liang J, Zhao G Elevated serum ferritin concentrations in a glucose-impaired population and in normal glucose tolerant first-degree relatives in familial type 2 diabetic pedigrees. Diabetes Care 2004; 27(2): 622–3. doi: 10.2337/diacare.27.2.622. 14747258

[23] Jehn M, Clark JM, Guallar E Serum ferritin and risk of the metabolic syndrome in U.S. adults. Diabetes Care 2004; 27(10): 2422–8. doi: 10.2337/diacare.27.10.2422. 15451911

[24] Jiang L, Wang A, Molyneaux LM, Constantino MI, Yue DK The long-term impact of ferritin level on treatment and complications of type 2 diabetes. Diabetes Obes Metab 2008; 10(6): 519–22. doi: 10.1111/j.1463-1326.2008.00855.x. 18462199

[25] Fernandez-Real JM, Penarroja G, Castro A, Garcia-Bragado F, Hernandez-Aguado I, Ricart W Blood letting in high-ferritin type 2 diabetes: effects on insulin sensitivity and beta-cell function. Diabetes 2002; 51(4): 1000–4. doi: 10.2337/diabetes.51.4.1000. 11916918

[26] Qian M, Liu M, Eaton JW Transition metals bind to glycated proteins forming redox active “glycochelates”: implications for the pathogenesis of certain diabetic complications. Biochem Biophys Res Commun 1998; 250(2): 385–9. doi: 10.1006/bbrc.1998.9326. 9753639

[27] Wirfalt E, Hedblad B, Gullberg B, Mattisson I, Andren C, Rosander U, et al Food patterns and components of the metabolic syndrome in men and women: a cross-sectional study within theMalmö Diet and Cancer cohort. Am J Epidemiol 2001; 154(12): 1150–9. doi: 10.1093/aje/154.12.1150. 11744521

[28] Wittenbecher C, Muhlenbruch K, Kroger J, Jacobs S, Kuxhaus O, Floegel A, et al Amino acids, lipid metabolites, and ferritin as potential mediators linking red meat consumption to type 2 diabetes. Am J Clin Nutr 2015; 101(6): 1241–50. doi: 10.3945/ajcn.114.099150. 25948672

[29] Cooksey RC, Jouihan HA, Ajioka RS, Hazel MW, Jones DL, Kushner JP, et al Oxidative stress, beta-cell apoptosis, and decreased insulin secretory capacity in mouse models of hemochromatosis. Endocrinology 2004; 145(11): 5305–12. doi: 10.1210/en.2004-0392 15308612

[30] Niederau C, Berger M, Stremmel W, Starke A, Strohmeyer G, Ebert R, et al Hyperinsulinaemia in non-cirrhotic haemochromatosis: impaired hepatic insulin degradation? Diabetologia 1984; 26(6): 441–4. doi: 10.1007/BF00262217 6381191

[31] Ferrannini E Insulin resistance, iron, and the liver. Lancet 2000; 24; 355(9222): 2181–2. doi: 10.1016/S0140-6736(00)02397-7. 10881887

[32] Yao D, Shi W, Gou Y, Zhou X, Yee Aw T, Zhou Y, et al Fatty acid-mediated intracellular iron translocation: a synergistic mechanism of oxidative injury. Free Radic Biol Med 2005; 39(10): 1385–98. doi: 10.1016/j.freeradbiomed.2005.07.015. 16257648

[33] Savolainen O, Lind MV, Bergstrom G, Fagerberg B, Sandberg AS, Ross A Biomarkers of food intake and nutrient status are associated with glucose tolerance status and development of type 2 diabetes in older Swedish women. Am J Clin Nutr 2017; 106(5): 1302–10. doi: 10.3945/ajcn.117.152850. 28903960

[34] Li K, Wu K, Zhao Y, Huang T, Lou D, Yu X, et al Interaction between marine- derived n-3 long chain polyunsaturated fatty acids and uric acid on glucose metabolism and risk of type 2 diabetes mellitus: a case-control study. Mar Drugs 2015; 13(9): 5564–78. doi: 10.3390/md13095564. 26343686 PMC4584340

[35] Lapolla A, Sartore G, Della Rovere GR, Romanato G, Zambon S, Marin R, et al Plasma fatty acids and lipoproteins in type 2 diabetic patients. Diabetes Metab Res Rev 2006; 22(3): 226–31. doi: 10.1002/dmrr.607. 16308886

[36] Sun Q, Ma J, Campos H, Hankinson SE, Hu FB Comparison between plasma and erythrocyte fatty acid content as biomarkers of fatty acid intake in US women. Am J Clin Nutr. 2007; 86(1): 74–81. doi: 10.1093/ajcn/86.1.74. 17616765

[37] Jo S, An WS, Park Y Erythrocyte n-3 polyunsaturated fatty acids and the risk of type 2 diabetes in Koreans: a case-control study. Ann Nutr Metab 2013; 63(4): 283–90. doi: 10.1159/000357018. 24334969

[38] Lee C, Liese A, Wagenknecht L, Lorenzo C, Haffner S, Hanley A Fish consumption, insulin sensitivity and beta-cell function in the Insulin Resistance Atherosclerosis Study (IRAS). Nutr Metab Cardiovasc Dis 2013; 23(9): 829–35. doi: 10.1016/j.numecd.2012.06.001. 22835984 PMC3485446

[39] Flachs P, Rossmeisl M, Kopecky J The effect of n-3 fatty acids on glucose homeostasisand insulin sensitivity. Physiol Res 2014; 63(Suppl 1): S93–118. doi: 10.1186/s12944-017-0528-0. 24564669

[40] Alhassan A, Young J, Lean MEJ, Lara J Consumption of fish and vascular risk factors: a systematic review and meta-analysis of intervention studies. Atherosclerosis 2017; 266: 87–94. doi: 10.1016/j.atherosclerosis.2017.09.028. 28992469

[41] Larsson SC, Orsini N Fish consumption and the risk of stroke: a dose-response meta-analysis. Stroke 2011; 42(12): 3621–3. doi: 10.1161/STROKEAHA.111.630319. 21903950

[42] Djousse L, Akinkuolie AO, Wu JH, Ding EL, Gaziano JM Fish consumption, omega-3 fatty acids and risk of heart failure: a meta-analysis. Clin Nutr 2012; 31(6): 846–53. doi: 10.1016/j.clnu.2012.05.010. 22682084 PMC3509256

[43] Zheng J, Huang T, Yu Y, Hu X, Yang B, Li D Fish consumption and CHD mortality: an updated meta-analysis of seventeen cohort studies. Public Health Nutr 2012; 15(4): 725–37. doi: 10.1017/S1368980011002254. 21914258

[44] Wheeler ML, Dunbar SA, Jaacks LM, Karmally W, Mayer-Davis EJ, Wylie- Rosett J, et al Macronutrients, food groups, and eating patterns in the management of diabetes: a systematic review of the literature, 2010. Diabetes Care 2012; 35(2): 434–45. doi: 10.2337/dc11-2216. 22275443 PMC3263899

[45] Belalcazar LM, Reboussin DM, Haffner SM, Reeves RS, Schwenke DC, Hoogeveen RC, et al Marine omega-3 fatty acid intake: associations with cardiometabolic risk and response to weight loss intervention in the Look AHEAD (Action for Health in Diabetes) study. Diabetes Care 2010; 33(1): 197–9. doi: 10.2337/dc09-1235. 19841042 PMC2797972

[46] Evert AB, Boucher JL, Cypress M, Dunbar SA, Franz MJ, Mayer-Davis EJ, et al Nutrition therapy recommendations for the management of adults with diabetes. Diabetes Care. 2014; 37(Suppl 1): S120–43. doi: 10.2337/dc14-S120. 24357208

[47] Akbari M, Tabrizi R, Lankarani KB, Heydari ST, Karamali M, Kashanian M, et al. The Effects of folate supplementation on diabetes biomarkers among patients with metabolic diseases: a systematic review and meta-analysis of randomized controlled trials. Horm Metab Res. 2018;50(2):93–105. doi: 10.1055/s-0043-125148. 29342488

