# Supplementary material for: Blood biomarkers of various dietary patterns correlated with metabolic indicators in Taiwanese type 2 diabetes
Source: Food Nutr Res. 2019 Nov 20;63:10.29219/fnr.v63.3592. doi: 10.29219/fnr.v63.3592 (PMC6878969; doi:10.29219/fnr.v63.3592)
Supplement: Blood biomarkers of various dietary patterns correlated with metabolic indicators in Taiwanese type 2 diabetes [file FNR-63-3592-s001.docx]

| **Supplementary Table 1**. Factor-loading matrix for the three dietary patterns identified from the food frequency questionaires (FFQ) by factor analysis in type 2 diabetic patients (n=871). | | | |
| --- | --- | --- | --- |
| Food item | Dietary patterns | | |
|  | High fat-meat | Traditional Chinese food-snack | Fish-vegetable |
| White meats (T/W) | 0.667 | － | － |
| Fatty meats and skin (T/W) | 0.636 | － | － |
| Fried food and fried snacks (T/W) | 0.611 | 0.222 | － |
| Seafood (T/W) | 0.599 | － | － |
| Red meats (T/W) | 0.583 | － | － |
| Smoked and processed meats (T/W) | 0.537 | － | － |
| Eating out (T/W) | 0.510 | － | 0.241 |
| Egg (P/W) | 0.367 | － | － |
| Tea (1 serving=240c.c.) (P/W) | 0.335 | 0.222 | － |
| Chinese staple food (1 portion=1 bowl) (P/W) | 0.308 | － | － |
| Canned meats (T/W) | 0.268 | 0.253 | － |
| Sweetened drinks (1 serving=240c.c.) (P/W) | 0.261 | 0.219 | － |
| Wheat processed products/gluten products (T/W) | － | 0.621 | － |
| Soybean products (T/W) | － | 0.601 | － |
| Root vegetables (B/W) | － | 0.440 | － |
| Starchy/thicken soup and food (T/W) | 0.216 | 0.424 | 0.210 |
| Pickled vegetables (T/W) | － | 0.400 | 0.216 |
| Low nitrogen staple food (T/W) | 0.273 | 0.380 | － |
| Low calories dessert (T/W) | 0.254 | 0.368 | － |
| Nuts (T/W) | 0.208 | 0.333 | 0.234 |
| Cake and cookie (T/W) | － | 0.272 | － |
| Sugar substitute use (T/W) | － | 0.264 | － |
| Sweetened fruit juice (T/W) | － | 0.245 | － |
| Dairy processed products (P/W) | － | 0.237 | － |
| Light-colored vegetables (T/W) | － | 0.221 | 0.689 |
| Dark-colored vegetables (T/W) | － | 0.262 | 0.671 |
| Marine fish (T/W) | 0.347 | -0.246 | 0.639 |
| Freshwater aquaculture fish (T/W) | 0.342 | -0.364 | 0.515 |
| Fresh fruit (P/W) | － | － | 0.284 |
| Milk, yogurt drink, or goat milk (P/W) | － | － | 0.253 |
| Food with a factor loading < ±0.2 are not shown.  T/W: Time/week; P/W: Portion/week; B/W: Bowl/week. | | | |
